# Genetic analysis of neurodevelopmental disorders in children

**DOI:** 10.3389/frcha.2022.987339

**Published:** 2022-12-07

**Authors:** Dandan Wu, Rong Li

**Affiliations:** Child Healthcare Department, Children's Hospital of Nanjing Medical University, Nanjing, China

**Keywords:** neurodevelopmental disorders, global developmental/intellectual disabilities, autism spectrum disorders, children, gene

## Abstract

**Purpose:**

To explore the genetic cause of children with unidentified etiology of neurodevelopmental disorders, thus providing references for the diagnosis, treatment and genetic counseling.

**Design and methods:**

Children with neurodevelopmental disorders but unidentified etiology in the Child Healthcare Department, Children's Hospital of Nanjing Medical University from November 2018 to December 2021 were retrospectively analyzed. A total of 2 ml of peripheral venous blood was collected from the child and their parents for the whole exome sequencing (WES) and copy number variation (CNV) detection. Male children were subjected to fragile X syndrome testing to determine the genetic mutations. For those with positive results, Sanger sequencing was performed to explore the mutations in the gene sites and pedigrees.

**Results:**

A total of 488 (33.5%) pathogenic variations were detected among 1,457 global developmental/intellectual disabilities (GDD/ID) children, including 362 (24.9%) cases of monogenic mutations, and 111 (7.6%) cases of chromosomal microdeletions or microduplications. There were 15/780 (1.92%) male children with fragile X syndrome. Single point mutations were detected in 277/362 (76.5%) and 85/362 (23.5%) male and female GDD/ID children, respectively, including 295 (81.5%) cases of missense mutations, 32 (8.8%) cases of frameshift mutations, 5 (2.2%) cases of non-sense mutations and 30 (8.3%) cases of splice site mutations. In addition, there were 166 (45.8%) cases of autosomal inheritance and 196 (54.2%) cases of X-linked inheritance. The X chromosome abnormalities were mostly observed in 362 GDD/ID children with monogenic mutations, including 15 cases of the *AFF2* gene mutation, 13 cases of the *MECP2* gene mutation and 12 cases of the *HUWEI* gene mutation. The *CREBBP* gene mutation was the most common autosome abnormality in GDD/ID children with monogenic mutations, which was detected in five cases. There were 74 cases of chromosomal microdeletions, 31 cases of chromosomal microduplications and six cases of both. A total of 114 novel pathogenic mutations responsible for GDD/ID were found, including four novel mutations in *MECP2* and *TRAPPC9* genes.

**Conclusion:**

The incidence of genetic abnormalities remains high in NDD children. Abundant novel mutations are responsible for GDD/ID in children, and can be used as references in the diagnosis of neurogenetic diseases.

## Introduction

Neurodevelopmental disorders (NDD), an array of multifactorial chronic diseases, seriously impair children's physical and psychological health, even cross their lifespan. NDD include at least global developmental/intellectual disabilities (GDD/ID), autism spectrum disorders (ASD), both as main contributors to pediatric disability worldwide. GDD/ID exhibit highly heterogeneous clinical and genetic features, often complicated with ASD and attention deficit and hyperactivity disorder (ADHD) [1]. ID has a global prevalence of 1%, and serious ID of 0.6%. ASD was first reported in 1943, with a global incidence ever-growing to 1% in 2012 according to the WHO data [2–4].

NDD are caused by environmental and genetic factors. In an era with updating livelihood and enriching medical resources, exogenous factors, like infection, poisoning, trauma, hypoxia, malnutrition, illiteracy, psychological impairment, have been well-uncovered and controlled. In contrast, genetic factors remain enigmatic by large. About 2/3 of ID are caused by genetic factors, including chromosome abnormality, single or multiple-gene mutation, or inborn errors of metabolism. With plural manifestations, ID meets with no effective treatments, leading to a high rate of disability. Special education or rehabilitation training may alleviate ID symptoms, but their application across the lifespan is restricted by a huge cost [5–8].

Genetic diagnosis and counseling can benefit the treatment of NDD children, even prevent secondary adverse pregnancy outcomes. In the present study, a total of 1,457 GDD/ID and ASD children with unidentified etiology were collected and analyzed with the whole exome sequencing (WES). For those with positive results, Sanger sequencing was performed to explore the mutations in the gene sites and pedigrees. Male children were subjected to fragile X syndrome testing to determine the genetic mutations. Our findings are expected to provide references for the diagnosis and treatment of children with GDD/ID or ASD.

## Materials and methods

### Setting and participants

We recruited 1,457 children treated for GDD/ID and ASD of unclear etiology from November 2018 to December 2021 at the Department of Children Health Care, Children's Hospital of Nanjing Medical University (Nanjing, China), including 1,152 males (79.1%) and 305 females (20.9%). Inclusion criteria were as follows: (1) Assessment of GDD/ID. Gesell Developmental Scales (Gesell) [9], Griffiths Development Scales-Chinese Edition (GDS-C) [10], Wechsler Preschool and Primary Scale of Intelligence (WPPSI-IV) [11], Wechsler Intelligence Scale for Children (WISC-IV) [12], and Infants-Junior Middle School Student's Social-Life Abilities Scale (S-M scale) [13] were adopted to assess GDD/ID in children. Children with two or more domains of Gesell and GDS-C that were significantly lower than those in children of the same age (>2 standard deviations) were assessed as GDD. WPPSI-IV and WISC-IV were used to assess GDD/ID in children with 2.5–6 and 6–16 years, respectively. According to the intelligence quotient (IQ) range and social adaptability, ID was classified into mild (IQ: 50–69), moderate (IQ: 35–49), severe (IQ: 20–34), and profound (IQ <20). Corresponding developmental assessment scales were used according to the developmental level and age of children (Table 1). (2) Assessment of ASD. The Autism Behavior Checklist (ABC) [14] and the Childhood Autism Rating Scale (CARS) [15] were adopted to assess autism symptoms in children. ASD was diagnosed in children with the total ABC score ≥67 points and CARS score ≥30 points.

**Table 1:** Corresponding developmental assessment scales for GDD/ID children at different ages.

**Age**	**Developmental assessment scales**
0–3 years	Gesell
3–6 years	GDS-C (non-verbal children)
	WPPSI-IV+ S-M scale
6–8 years	GDS-C (non-verbal children)
	WISC-IV+ S-M scale
>8 years	WISC-IV + S-M scale

GDD/ID, global development disabilities/intellectual disabilities; Gesell, Gesell Developmental Scales; GDS-C, Griffiths Development Scales-Chinese Edition; WPPSI-IV, Wechsler Preschool and Primary Scale of Intelligence; S-M scale, Infants-Junior Middle School Student's Social-Life Abilities Scale; WISC-IV, Wechsler Intelligence Scale for Children.

Exclusion criteria: (1) children with specific genetic diseases and genetic metabolic diseases diagnosed by clinical symptoms and signs, and laboratory testing; (2) children with GDD/ID and ASD caused by perinatal ischemia, hypoxic brain injury, bilirubin encephalopathy, and central nervous system infection, poisoning and trauma.

### Procedures

After obtaining the written informed consent of the guardians, 2 ml of peripheral venous blood was collected from each child and his/her parents for WES and CNV detection. Male children were subjected to fragile X syndrome testing to determine the genetic mutations. For those with positive results, Sanger sequencing was performed to explore the mutations in the gene sites and pedigrees.

#### Target capture sequencing (TCS)

DNA fragments of the target region were enriched by microarray hybridization, then subjected to the next-generation high-throughput sequencing. In the present study, the exon regions of 23,000 genes were captured using the Gen Cap custom exome enrichment kit (My Genostics, Beijing, China). Briefly, genomic DNA fragments were ligated to the Illumina-supplied adapter sequences, then subjected to ligation-mediated polymerase chain reaction (LM-PCR). After amplification and purification, the quality of DNA library was checked. PCR products were hybridized with the target capture microarray, and the captured sequence was analyzed using the Illumina Nova 6000 sequencer (Illumina, San Diego, CA, USA). Raw data were processed for image recognition and differentiation.

#### Bioinformatic analyses

After trimming adapter sequences from short read data, the Illumina sequencing reads were mapped to the human reference genome (GRCh37/hg19, 2009 Assembly) using the BWA-MEM alignment algorithm of Burrows-Wheeler Aligner (BWA, http://bio-bwa.sourceforge.net) [16, 17]. Single nucleotide variation (SNV) and inserts and deletions (INDEL) were analyzed using the Genome Analysis Toolkit (GATK, http://software.broadinstitute.org) [18], and annotated by ANNOVAR (http://annovar.openbioinformatics.org/en/latest/) [19]. Mutation sites with a frequency of <0.05 were screened out of the following databases, including the 1,000 Genomes Project (http://www.1000genomes.org), Exome Variant Server (http://evs.gs.washington.edu) and EXAC (http://exac.broadinstitute.org/). Pathogenicity and conservative prediction of missense mutations were carried out using SIFT (http://sift.jcvi.org/) [20–22], PolyPhen-2 (http://genetics.bwh.harvard.edu/pph2/) [23–25], MutationTaster (http://www.mutationtaster.org/) [26], and GERP++ (http://mendel.stanford.edu/SidowLab/downloads/gerp/index.html) [27]. Changes in splice sites were analyzed for pathogenicity using SPIDEX (http://www.openbioinformatics.org/annovar/spidex_download_form.php).

#### PCR and sanger sequencing

Mutations of interest were subjected to PCR and Sanger sequencing. PCR primers were synthesized using Primer 3.0 (http://primer3.ut.ee/) [28–30]. In addition, PCR products were subjected to Sanger sequencing and analyzed using the ABI 3130 Genetic Analyzer (Applied Biosystems), followed by the family cosegregation analysis.

#### Fragile X syndrome testing

DNA extracted from peripheral blood was analyzed by PCR, and the PCR fragment was designed to flank the area of repeats and effectively adjust the size of the PCR product correspondingly to the repeat size. After allele-specific methylation PCR, capillary electrophoresis was performed to measure the number of CTG repeats.

### Statistical analysis

Data were expressed as medians and interquartile ranges (IQRs), and processed using SPSS 22.0. *P* < 0.05 was considered as statistically significant.

## Results

### Types of pathogenic variations

There were 1,253/1,457 (85.9%) children with GDD/ID and 204/1,457 (14.1%) with GDD/ID and autism. A total of 488 (33.5%) pathogenic variations were detected among 1,457 GDD/ID children, including 362 (24.9%) monogenic mutations, and 111 (7.6%) chromosomal microdeletions or microduplications. Fifteen male children (1.92%, 15/780) aged 2–13 years presented with fragile X syndrome, and four of their mothers were determined as premutation carriers, while *FMR1* premutation was not detected in their fathers. Notably, full mutation in *FMR1* gene was detected in 9 male children of 4 pedigrees (Table 2).

**Table 2:** Baseline characteristics of GDD/ID children.

**Characteristics**	**Number of patients (%)**
Total	1,457
Sex	
Male	1,152 (79.1%)
Female	305 (20.9%)
Age	
≤ 1 year	402 (27.6%)
>1 year, ≤ 3 years	753 (51.7%)
>3 year, ≤ 6 years	231 (15.9%)
>6 year	71 (4.8%)
Genetic etiology	
CGG repeat expansion in the *FMR1* gene	15 (1.0%)
Small pathogenic variations	362 (24.9%)
Pathogenic CNVs	111 (7.6%)
Total	488 (33.5%)

GDD/ID, global development disabilities/intellectual disabilities; CNV, copy number variation.

### Monogenic mutations in GDD/ID children

#### Common monogenic mutations responsible for GDD/ID

Most of the X chromosome abnormalities were observed in 362 GDD/ID children with monogenic mutations, including 15 with *AFF2* gene mutation, 13 with *MECP2* gene mutation and 12 with *HUWEI* gene mutation. The *CREBBP* gene mutation, detected in five cases, was the most common autosome abnormality in GDD/ID children with monogenic mutations (Table 3).

**Table 3:** Pathogenic variations in GDD/ID children.

**Gene**	**Inheritance pattern**	**Number of patients (%)**	**Number of male patients (%)**	**Number of female patients (%)**
*AFF2*	XLR	15 (1.03%, 15/1,457)	13 (1.13%, 13/1,152)	2 (0.66%, 2/305)
*MECP2*	XL	13 (0.89%, 13/1,457)	6 (0.52%, 6/1,152)	7 (2.23%, 7/305)
*HUWEI*	XL	12 (0.82%, 12/1,457)	11 (0.95%, 11/1,152)	1 (0.33%, 1/305)
*BRWD3*	XLR	10 (0.69%, 10/1,457)	11 (0.95%, 11/1,152)	0
*ATRX*	XL	6 (0.4%, 6/1,457)	5 (0.43%, 5/1,152)	1 (0.33%, 1/305)
*DLG3*	XLR	5 (0.34%, 5/1,457)	5 (0.43%, 5/1,152)	0
*ZNF41*	XL	5 (0.34%, 5/1,457)	5 (0.43%, 5/1,152)	0
*PTCHD1*	XLR	5 (0.34%, 5/1,457)	5 (0.43%, 5/1,152)	0
*CREBBP*	AD	5 (0.34%, 3/1,457)	3 (0.26%, 3/1,152)	2 (0.66%, 2/305)

GDD/ID, global development disabilities/intellectual disabilities; XLR, X-linked recessive inheritance; XL, X-linked inheritance; AD, autosomal dominant inheritance.

#### Single point mutations in GDD/ID children

Monogenic mutations were detected in 362 GDD/ID children, involving 277 males and 85 females. Among them, 295 carried missense mutations, 32 carried frameshift mutations, five carried non-sense mutations, and 30 carried splice site mutations (Table 4). In particular, non-sense mutations were inherited in 3 GDD/ID children with an autosomal recessive pattern, involving 1 combined with splice site mutation and 2 with frameshift mutations.

**Table 4:** Single site mutations in GDD/ID children.

**Mutation type**	**Number of patients (%)**	**Number of male patients (%)**	**Number of female patients (%)**
Missense mutation	295 (81.5%, 295/362)	237 (85.6%, 237/277)	58 (68.2%, 58/85)
Frameshift mutation	32 (8.8%, 32/362)	16 (5.6%, 16/277)	16 (18.8%, 16/85)
Nonsense mutation	5 (1.4%, 5/362)	0 (0%, 0/277)	5 (5.9%, 5/85)
Splice site mutation	30 (8.3%, 30/362)	24 (8.7%, 24/277)	6 (7.1%, 6/85)

GDD/ID, global development disabilities/intellectual disabilities.

#### Novel single point mutations in GDD/ID children

Newly discovered single point mutations were abundant in 362 cases of GDD/ID children, all never reported before. The mutations were considered pathogenic (**P**) based on protein damage due to mutation-induced premature termination codon and clinical manifestations of affected children. Moreover, their pathogenicity (p) was considered as positive results from more than four online tools in predicting the pathogenicity of rare missense variants (e.g., SIFT, PolyPhen_2MutationTaster, GERP++, and REVEL). At last, 114 novel pathogenic mutations responsible for GDD/ID were found (Table 5).

**Table 5:** Novel single point mutations in GDD/ID children.

**Patient ID**	**Gene**	**Inheritance pattern**	**CDS change**	**Amino-acid change**	**Zygosity**	**Patho-genicity**	**Origi-n**
Case 1	*POMT1*	AR	NM_007171 c.1624T>G	p.F542V	het^a^	P^h^	Mat^f^
	*POMT1*	AR	NM_007171 c.1901_1902insGCG CTGGGTGCTGGC TGGG	p.C643Gfs*94	het^a^	**P** ^g^	Dn^d^
Case 2	*ZSWIM6*	AD	NM_020928 c.1033+1G>T	Splicing	het^a^	P^h^	Dn^d^
Case 3	*DCX*	XL	NM_000555 c.982G>T	p.D328Y	hemi^b^	P^h^	Mat^f^
Case 4	*THOC2*	XLR	NM_001081550 c.4237G>T	p.D1413Y	hemi^b^	P^h^	Mat^f^
Case 5	*MBD5*	AD	NM_018328 c.1472G>T	p.R491M	het^a^	P^h^	Mat^f^
Case 6	*OTC*	XLR	NM_000531 c.1024C>A	p.L342M	hemi^b^	P^h^	Mat^f^
Case 7	*DLG3*	XLR	NM_021120 c.2351T>C	p.I784T	hemi^b^	P^h^	Mat^f^
Case 8	*MAN2B1*	AR	NM_000528 c.2993G>A	p.R998H	het^a^	P^h^	Mat^f^
	*MAN2B1*	AR	NM_000528 c.748G>T	p.A250S	het^a^	P^h^	Pat^e^
Case 9	*SMC1A*	XLD	NM_006306 c.673C>T	(p.L225F)	hemi^b^	P^h^	Mat^f^
Case 10	*PRSS12*	AR	NM_003619 c.905delG	p.G302fs*15	het^a^	**P** ^g^	Mat^f^
	*PRSS12*	AR	NM_003619 c.2476G>A	p.G826R	het^a^	P^h^	Pat^e^
Case 11	*IL1RAPL1*	XLR	NM_014271 c.1033G>A	p.A345T	hemi^b^	P^h^	Mat^f^
Case 12	*HUWE1*	XL	NM_031407 c.10930G>A	(p.E3644K)	hemi^b^	P^h^	Mat^f^
Case 13	*STAG2*	XL	NM_001042749 c.403A>G	(p.M135V)	hemi^b^	P^h^	Mat^f^
Case 14	*SCN8A*	AD	NM_014191 c.2130A>C	(p.E710D)	het^a^	P^h^	Mat^f^
Case 15	*CASK*	XLD	NM_003688 c.62G>A	(p.G21D)	hemi^b^	P^h^	Mat^f^
Case 16	*SMC1A*	XLD	NM_006306 c.3362G>A	p.R1121H	het^a^	P^h^	Dn^d^
Case 17	*DYNC1H1*	AD	NM_001376 c.5684C>A	(p.A1895D)	het^a^	P^h^	Dn^d^
Case 18	*FBXO11*	AD	NM_001190274 c.1421T>G	(p.I474R)	het^a^	P^h^	Dn^d^
Case 19	*CAMK2A*	AD	NM_015981 c.74C>T	p.S25L	het^a^	P^h^	Dn^d^
Case 20	*HUWE1*	XL	NM_031407 c.1708C>A	p.P570T	hemi^b^	P^h^	Dn^d^
Case 21	*SCN2A*	AD	NM_021007 c.809T>G	p.F270C	het^a^	P^h^	Dn^d^
Case 22	*CTCF*	AD	NM_006565 c.950C>G	(p.T317R)	het^a^	P^h^	Dn^d^
Case 23	*SATB2*	AD	NM_001172509, c.1942C>T	p.L648F	het^a^	**P** ^g^	Dn^d^
Case 24	*HCN1*	AD	NM_021072 c.1082A>G	p.Y361C	het^a^	**P** ^g^	Dn^d^
Case 25	*GRIN2B*	AD	NM_000834 c.2108A>C	p.H703P	het^a^	**P** ^g^	Dn^d^
Case 26	*SMC3*	AD	NM_005445 c.3520A>G	p.T1174A	het^a^	**P** ^g^	Dn^d^
Case 27	*MECP2*	XLD	NM_004992 c.397C>T	p.R133C	hemi^b^	**P** ^g^	Dn^d^
Case 28	*MECP2*	XL	NM_001110792 c.916C>T	p.R306X	hemi^b^	**P** ^g^	Dn^d^
Case 29	*CREBBP*	AD	NM_004380 c.4883A>G	p.H1628R	het^a^	**P** ^g^	Dn^d^
Case 30	*PCNT*	AR	NM_006031 c.8997-1G>A	-	hom^c^	**P** ^g^	Mat^f^+Pat^e^
Case 31	*TCF4*	AD	NM_001083962 c.1727G>A	p.R576Q	het^a^	**P** ^g^	Dn^d^
Case 32	*IGF1R*	AR	NM_000875 c.1949G>A	p.R650Q	het^a^	P^h^	Mat^f^
	*IGF1R*	AR	NM_000875 c.2894G>A	p.S965N	het^a^	P^h^	Pat^e^
Case 34	*KMT2A*	AD	NM_001197104 c.3634+1G>C	-	het^a^	**P** ^g^	Dn^d^
Case 35	*PAFAH1B1*	AD	NM_000430 c.193-2A>T	-	het^a^	**P** ^g^	Dn^d^
Case 36	*DDX3X*	XL	NM_001193416 c.1582C>T	p.R528C	hemi^b^	**P** ^g^	Dn^d^
Case 37	*TRIO*	AD	NM_007118 c.4024G>A	p.G1342R	het^a^	**P** ^g^	Dn^d^
Case 38	*PPP2R1A*	AD	NM_014225 c.656C>T	p.S219L	het^a^	**P** ^g^	Dn^d^
Case 39	*SLC6A19*	AR	NM_001003841 c.985G>A	p.A329T	het^a^	P^h^	Mat^f^
	*SLC6A19*	AR	NM_001003841 c.1213G>A	p.E405K	het^a^	P^h^	Pat^e^
Case 40	*CTCF*	AD	NM_006565 c.1102C>T	p.R368C	het^a^	**P** ^g^	Dn^d^
Case 41	*KCNQ3*	AD	NM_004519 c.689G>A	p.R230H	het^a^	**P** ^g^	Dn^d^
Case 42	*DLG3*	XLR	NM_020730 c.58delT	p.S20Pfs*45	hemi^b^	**P** ^g^	Mat^f^
Case 43	*CC2D1A*	AR	NM_017721 c.1941-5G>A	Splicing	het^a^	P^h^	Mat^f^
	*CC2D1A*	AR	NM_017721 c.2342G>T	p.G781V	het^a^	P^h^	Pat^e^
Case 44	*BRWD3*	XLR	NM_153252 c.4591G>A	p.G1531R	hemi^b^	P^h^	Mat^f^
Case 45	*KDM5C*	XLR	NM_004187 c.1843G>C	p.V615L	hemi^b^	P^h^	Mat^f^
Case 46	*MECP2*	XL	NM_004992 c.378-8_378- 19delTTTCT GTTTGTC	Splicing	hemi^b^	P^h^	Mat^f^
Case 47	*NLG N4X*	XL	NM_020742 c.1612C>A	p.Q538K	hemi^b^	**P** ^g^	Mat^f^
Case 48	*AFF2*	XLR	NM_0011 70628 c.26C>T	p.A9V	hemi^b^	**P** ^g^	Mat^f^
Case 49	*SHANK2*	XL	NM_133266 c.1651G>A	p.E551K	het^a^	**P** ^g^	Mat^f^
Case 50	*ASXL3*	AD	NM_030632 c.1533_1534del	p.L512Afs*4	het^a^	**P** ^g^	Dn^d^
Case 51	*SHROOM4*	XL	NM_020717 c.722G>A	p.R241Q	hemi^b^	**P** ^g^	Mat^f^
Case 52	*PGAP2*	AR	NM_001256240 c.493C>T	p.R165W	het^a^	**P** ^g^	Dn^d^
	*PGAP2*	AR	NM_001256240 c.590C>T	p.A197V	het^a^	P^h^	Mat^f^
Case 53	*TRAPPC9*	AR	NM_031466 c.2314T>A	p.L772M	het^a^	**P** ^g^	Pat^e^
	*TRAPPC9*	AR	NM_001160372 c.1981+3A>G	Splicing	het^a^	**P** ^g^	Mat^f^
Case 54	*THOC2*	XLR	NM_001081550 c.275-7A>G	Splicing	hemi^b^	**P** ^g^	Mat^f^
Case 55	*TINF2*	AD	NM_001099274 c.953_956delTGG GinsCGG	p.MG318Tfs	het^a^	**P** ^g^	Pat^e^
Case 56	*FOXRED1*	AR	NM_017547 c.608_609delAG	p.E203fs*16	het^a^	**P** ^g^	Pat^e^
	*FOXRED1*	AR	NM_017547 c.395C>T	p.A132V	het^a^	**P** ^g^	Mat^f^
Case 57	*HUWE1*	XL	NM_031407 c.5521-6T>G	Splicing	hemi^b^	**P** ^g^	Mat^f^
Case 58	*MED12*	XLR	NM_005120 c.4416-3T>C	Splicing	hemi^b^	**P** ^g^	Mat^f^
Case 59	*NLGN4X*	XL, IC, Mu	NM_020742 c.2104_2105dupAA	p.R704fs*32	hemi^b^	**P** ^g^	Mat^f^
Case 60	*BPTF*	AD	NM_004459 c.4413_4414delTG	p.C1471X	het^a^	**P** ^g^	Mat^f^
Case 61	*IQSEC2*	XLD	NM_015075 c.12dupC	p.G5Rfs*23	hemi^b^	**P** ^g^	Mat^f^
Case 62	*ASXL3*	AD	NM_030632 c.4798C>T	p.Q1600X	het^a^	**P** ^g^	Dn^d^
Case 63	*BRWD3*	XLR	NM_153252 c.3481+6A>G	Splicing	hemi^b^	**P** ^g^	Mat^f^
Case 64	*NHLRC2*	AR	NM_198514 c.950T>C	p.I317T	hom^c^	**P** ^g^	Mat^f^+Pat^e^
Case 65	*QRICH1*	AD	NM_017730 c.1740G>A	p.W580X	het^a^	**P** ^g^	Dn^d^
Case 66	*BCORL1*	XLR	NM_021946 c.4946C>T	p.T1649I	hemi^b^	**P** ^g^	Mat^f^
Case 67	*SOX4*	AD	NM_003107 c.309_c.311delCGA	p.D104del	het^a^	**P** ^g^	Dn^d^
Case 68	*KMT2C*	AD	NM_170606 c.5777_5778del	p.C1926Yfs*8	het^a^	**P** ^g^	Dn^d^
Case 69	*SHANK3*	AD	NM_033517 c.4727dupT	p.A1578Gfs*102	het^a^	**P** ^g^	Dn^d^
Case 70	*CTNNB1*	AD	NM_001904 c.1830delC	p.Q611Kfs*3	het^a^	**P** ^g^	Dn^d^
Case 71	*FLNA*	XLD	NM_001456 c.987+1G>A	Splicing	het^a^	**P** ^g^	Dn^d^
Case 72	*ZBTB18*	AD	NM_205768 c.583C>T	p.R195X	het^a^	**P** ^g^	Dn^d^
Case 73	*PPM1D*	AD	NM_003620 c.1303_1306dupCATA	p.I436Tfs*8	het^a^	**P** ^g^	Dn^d^
Case 74	*BCL11B*	AD	NM_138576 c.1946_1967delGCGC GGTCAACGGGCGC GGGGG	p.G649Afs*67	het	**P** ^g^	Dn^d^
Case 75	*SETD5*	AD	NM_001080517 c.675C>A	p.Y225X	het^a^	**P** ^g^	Dn^d^
Case 77	*DDX3X*	XLR, XLD	NM_001193416 c.1616-1G>T	Splicing	het^a^	**P** ^g^	Dn^d^
Case 78	*CREBBP*	AD	NM_004380 c.6324C>G	p.Y2108X	het^a^	**P** ^g^	Dn^d^
Case 79	*CD96*	AD	NM_198196 c.1006_1010delGTTTT	p.V336fs*5	het^a^	**P** ^g^	Dn^d^
Case 80	*AUTS2*	AD	NM_015570 c.1946G>A	p.W649X	het^a^	**P** ^g^	Dn^d^
Case 81	*DYNC1H1*	AD	NM_001376 c.4401_4405delAGAAG	p.R1467Sfs*6	het^a^	**P** ^g^	Dn^d^
Case 82	*GATAD2B*	AD	NM_020699 c.1195_1196del	p.Q399Efs*4	het^a^	**P** ^g^	Dn^d^
Case 83	*KMT2A*	AD	NM_001197104 c.502_502+1delGGinsC	Splicing	het^a^	**P** ^g^	Dn^d^
Case 84	*CTNNB1*	AD	NM_001904 c.1973dupT	p.L659Ffs*6	het^a^	**P** ^g^	Dn^d^
Case 85	*SHANK2*	-	NM_133266 c.3412_3413del	p.L1138V fs*16	het^a^	**P** ^g^	Dn^d^
Case 86	*SCN2A*	AD	NM_021007 c.5704delC	p.R1902Afs*20	het	**P** ^g^	Dn^d^
Case 87	*BRWD3*	XLR	NM_153252 c.4081-8_4081-6delCTT	Splicing	het^a^	**P** ^g^	Dn^d^
Case 88	*CACNA1C*	AD	NM_000719 c.396_399delGACT	p.T133Ffs*8	het^a^	**P** ^g^	Dn^d^
Case 89	*PHF21A*	AD	NM_001101802 c.1703delA	p.K568Sfs*9	het^a^	**P** ^g^	Dn^d^
Case 90	*ASXL2*	AD	NM_018263 c.2930_2934delTGAAA	p.M977Nfs*24	het^a^	**P** ^g^	Dn^d^
Case 91	*MED13*	AD	NM_005121 c.4552delT	p.S1518Qfs*16	het^a^	**P** ^g^	Dn^d^
Case 92	*FOXG1*	AD	NM_005249 c.896delT	p.F299Sfs*27	het^a^	**P** ^g^	Dn^d^
Case 93	*CREBBP*	AD	NM_004380 c.2608C>T	p.Q870X	het^a^	**P** ^g^	Dn^d^
Case 94	*ZNF292*	AD	NM_015021 c.4414C>T	p.R1472X	het^a^	**P** ^g^	Dn^d^
Case 95	*ANKRD11*	AD	NM_013275 c.4617_4618delGA	p.K1540Rfs* 13	het^a^	**P** ^g^	Dn^d^
Case 96	*CTCF*	AD	NM_006565 c.737delT	p.G247Vfs*2	het^a^	**P** ^g^	Dn^d^
Case 97	*HSD17B10*	XLD	NM_004493 c.748G>A	p.V250I	hemi^b^	P^h^	Mat^f^
Case 98	*TRAPPC9*	AR	NM_031466 c.1781C>T	p.S594L	het^a^	P^h^	Mat^f^
	*TRAPPC9*	AR	NM_031466 c.80C>G	p.S27C	het^a^	P^h^	Pat^e^
Case 99	*IQSEC2*	XLD	NM_001111125 c.1104G>T	p.E368D	hemi^b^	P^h^	Mat^f^
Case 100	*AFF2*	XLR	NM_002025 c.1358A>G	p.Q453R	hemi^b^	P^h^	Mat^f^
Case 101	*AFF2*	XLR	NM_002025 c.1084C>T	p.R362W	hemi^b^	P^h^	Mat^f^
Case 102	*MED12*	XLR	NM_005120 c.5584C>T	p.R1862C	hemi^b^	P^h^	Mat^f^
Case 103	*GRIA3*	XLR	NM_000828 c.425G>A	p.R142H	hemi^b^	P^h^	Mat^f^
Case 104	*NLGN3*	XL, IC, MU	NM_018977 c.290G>A	p.R97Q	hemi^b^	P^h^	Mat^f^
Case 105	*CACNA1A*	AD	NM-001127221 c.4989C>G	p.F1663L	het^a^	P^h^	Dn^d^
Case 106	*ZDHHC9*	XL	NM_016032 c.137T>G	p.L46R	hemi^b^	P^h^	Mat^f^
Case 107	*FTSJ1*	XLR	NM_012280 c.557G>A	p.R186Q	hemi^b^	P^h^	Dn^d^
Case 108	*SON*	AD	NM_032195 c.514A>G	p.R172G	het^a^	P^h^	Dn^d^

a het, heterozygosity.

b hemi, hemizygosity.

c homo, homozygosity.

d Dn, de novo.

e Pat, paternal.

f Mat, maternal.

g **P**, pathogenic.

h p, pathogenicity predicted to be positive in more than 4 online tools for predicting the pathogenicity of rare missense variants (e.g., SIFT, PolyPhen_2MutationTaster, GERP++, and REVEL).

GDD/ID, global development disabilities/intellectual disabilities; AR, autosomal recessive; AD, autosomal dominant inheritance; XL, X-linked inheritance; XLR, X-linked recessive inheritance; XLD, X-linked dominant inheritance.

#### Inheritance patterns of single point mutations in GDD/ID children

Among the 362 GDD/ID children with single point mutations, 166 (45.8%) presented with autosomal inheritance, and 196 (54.2%) presented with X-linked inheritance (Table 6).

**Table 6:** Inheritance patterns of the single site mutations in GD/IDD children.

**Inheritance pattern**	**Number of patients (%)**	**Number of male patients (%)**	**Number of female patients (%)**
AD	125 (34.5%, 125/362)	82 (30.7%, 85/277)	43 (50.6%, 43/85)
AR	41 (11.3%, 41/362)	27 (9.7%, 27/277)	14 (16.5%, 14/85)
XD	51 (14.1%, 51/362)	32 (11.6%, 32/277)	19 (22.4%, 19/85)
XR	145 (40.1%, 145/362)	136 (49.1%, 136/277)	9 (10.6%, 9/85)

GDD/ID, global development disabilities/intellectual disabilities; AD, autosomal dominant inheritance; AR, autosomal recessive; XD, X-linked dominant inheritance; XR, X-linked recessive inheritance.

#### Chromosomal microdeletions or microduplications

We identified 74 children with chromosomal microdeletions, 31 with chromosomal microduplications and 6 with both. Among the 111 (7.6%) cases of chromosomal microdeletions or microduplications, we detected 16 cases of Williams syndrome (WS), four cases of Phelan-McDermid syndrome (22q13 deletion syndrome), four cases of Prader-Willi syndrome or Angelman syndrome with deletions or duplications at the 15q11.2q13.1 locus (Table 7).

**Table 7:** Pathogenic CNVs in GD/IDD children.

	**Number of patients (%)**	**Number of male patients (%)**	**Number of female patients (%)**
Microdeletion	74 (66.7%, 74/111)	41 (74.5%, 41/55)	33 (58.9%, 33/56)
Microduplication	31 (27.9%, 31/111)	13 (23.6%, 13/55)	18 (32.1%, 18/56)
Microdeletion and microduplication	6 (5.4%, 6/111)	1 (1.8%, 1/55)	5 (8.9%, 5/56)

CNV, copy number variation; GDD/ID, global development disabilities/intellectual disabilities.

## Discussion

Clinical manifestations of NDD in children are diverse, the disability rate of which remains high. However, effective treatment for NDD lacks. Genetic factors are predominantly responsible for NDD, including chromosomal abnormalities, single or multiple gene mutations and congenital metabolic defects. In the present study, 362 (24.9%) pathogenic mutations were found in 1,457 GDD/ID children, dominated by missense mutation (295/362, 81.5%).

We found abundant single site mutations in GDD/ID children that have not been previously reported, and 114 of them were confirmed as pathogenic, including 51 (44.7%) of autosomal dominant, 21 (18.4%) of autosomal recessive, 8 (7.0%) of X-linked dominant, 18 (15.8%) of X-linked recessive and 16 (14.0%) of X-linked. Among them, 54 (47.4%) were inherited from parents, and 60 (52.6%) were spontaneous mutations. The latter was considered to pose a stronger destructive effect on biological functions than genetic mutations. Our findings provide valuable clinical evidence supporting the genetic causes of sporadic GDD/ID in children.

The newly discovered mutations in GDD/ID children have expanded the genetic spectrum of this disease. Notably, four novel mutations were found in *MECP2* and *TRAPPC9* genes, respectively. A total of seven female GDD/ID children carrying *MECP2* gene presented obvious intellectual disability and stereotyped behavior. *MECP2* gene is also the second most common gene for sex-linked genetic diseases. With the discovery of novel mutation sites, the incidence of neurodevelopmental disorders caused by *MECP2* gene is estimated to increase [31]. Novel mutations in the *TRAPPC9* gene were detected in 3 GDD/ID children. Notably, a GDD/ID child carried two novel mutations in the *TRAPPC9* gene with the clinical manifestations of severe intellectual disability and autism. NIBP/TRAPPC9 mutations are associated with non-syndromic autosomal recessive intellectual disability (NS-ARID), which is known as NIBP syndrome, and assigned as the intellectual disability-obesity-brain malformations facial dysmorphism syndrome in the human disease database Mala Cards [32].

All included GDD/ID children received WES and CNV detection. Chromosomal abnormalities and genomic microdeletions and microduplications act as critical genetic causes for NDD in children. In the present study, a total of 111 (7.6%) children with pathogenic CNVs were detected, involving 74 (66.7%) with microdeletions, 16 (27.9%) with microduplications and 6 (5.4%) with both. The incidence of microdeletions was much higher than that of microduplications in GDD/ID children. Theoretically, the incidence of genomic microdeletions is close to that of microduplications. In the early stage of gamete formation, however, neither duplication nor deletion is prone to occur. It is reported that the phenotype of microduplication is often milder (no clinical phenotype occurs) than that of microdeletion. Even at the chromosomal level, triploids are more likely to survive than haploids [33–35]. WS, a 7q11.23 duplication syndrome, was the most common disorder in GDD/ID children. Our findings differed from the previous studies of microdeletion or microduplication, which may be linked with the bigger data in our institution.

Sex-linked genetic diseases are characterized by an obvious between-sex difference. Healthy females can carry pathogenic genes of X-linked dominant and recessive disorders, bringing with a high risk of genetic diseases inherited through the pedigree. Among 1,457 GDD/ID children, we detected 196 (54.1%), 51 (14.1%), and 145 (40.1%) children X-linked inheritance, X-linked dominant inheritance, and X-linked recessive inheritance, respectively. The incidence of X-linked inheritance was similar to that of autosomal inheritance. However, X-linked recessive inheritance is more recessive and harmful. Chromosome positioning of 473 GDD/ID children carrying definite pathogenic genes showed that the X chromosome was the most common position of gene abnormalities. Consistently, the top 3 pathogenic genes (the *AFF2, MECP2*, and *HUWEI* gene) in GDD/ID children were also located in the X chromosome.

*AFF2* gene (OMIM 300806) maps to *Xq28* and is highly expressed in brain regions involved in learning, cognition, and memory, including the hippocampus and amygdala. Murine *AFF2* has an 88% amino acid similarity with human *AFF2*. *In situ* hybridization of adult mouse brains showed labeling in the cingulate gyrus, hippocampus, piriform cortex, and Purkinge layers [36, 37].

The expansion of CCG trinucleotide in the *AFF2* gene causes a form of X-linked intellectual disability (ID) related to Fragile site E (FRAXE) at Xq28. The clinical presentations of FRAXE fragility vary a lot, including mild intellectual disability, learning disabilities, communication deficits, attention deficit hyperactivity disorder (ADHD), and autistic spectrum. Translocations disrupting AFF2, partial or entire deletions and partial duplication have been described in ID patients. Partial AFF2 deletions, especially smaller deletions, are usually associated with a milder phenotype or autism, whereas complete loss of gene function causes FRAXE. A partial AFF2 duplication has been reported associated with mild ID [38–40].

In addition, Fragile X syndrome is inherited with an X-linked incomplete dominance, manifested as hereditary intellectual disability and autism. Mainly caused by the dynamic (CGG)n mutation, it develops with the silence or severe downregulation of Fragile X intellectual disability protein (FMRP). Fragile X syndrome is considered as the most common cause of intellectual disability in men in Europe and North America, which is observed in 10–20%. The prevalence of a full mutation of *FMR1* in the general population and females is 1/4,000–1/5,000 and 1/4,000–1/8,000, respectively. A large-scale screening of Fragile X syndrome in 51,000 newborns of mainland China in 2021 has shown that the frequencies of CGG repeats >100 in males and females are 1/9,371 and 1/5,887, respectively. In addition, the premutation rate of Fragile X syndrome in the Chinese population is lower than that in Caucasians [41]. In the present study, 15/780 (1.9%) of male GDD/ID children were diagnosed as Fragile X syndrome. Notably, the full mutation in the *FMR1* gene was detected in nine male children in four pedigrees.

Neurodevelopmental and psychiatric disorders are a highly heterogeneous group of developmental and mental disorders, resulting from the complex interaction between genetic and environmental factors and leading to various disabilities. The diagnosis of neurodevelopmental disorders is challenging, due to its multifactor, comorbidity and polygenicity. Its genetic mechanism has been firmly established, but the its causative variants remain to be discovered. At present, new tools, such as WES, have unearthed an increasing number of genetic variants responsible for human diseases.

In the present study, we performed the genetic analysis of neurodevelopmental disorders in 1,457 children from Jiangsu and Anhui Provinces, China, and provided etiological diagnosis and genetic consultation to 488 (33.5%) cases with identified pathogenic mutations. Our study provides references for prenatal diagnosis, thus preventing adverse reproductive outcomes. In addition, we found 114 novel pathogenic mutations responsible for GDD/ID, which expanded the genetic spectrum of GDD/ID. The *TRAPPC9* gene was not familiar with neurodevelopmental disorders, but showed 4 mutations in GDD/ID children. More neurodevelopment-associated loci in the *TRAPPC9* gene may be discovered in the future. In addition, the incidence of Fragile X syndrome in 488 children with identified pathogenic mutations was lower than those of *AFF2* (3.6%) and *HUWEI* (3.0%) in male GDD/ID children. Our findings are different from the previous that Fragile X syndrome is the second leading cause of male ID, which may be attributed to the genetic variations in different races. *AFF2* gene is closely linked with FRAXE, which was also the top pathogenic gene in the present study. Here, a total of 30 (2.1%) GDD/ID children presented both *AFF2* gene mutation and Fragile X syndrome. Sex-linked genetic diseases are the main cause of neurodevelopmental disorders in children, especially in males. Newborn screening of *AFF2* gene and Fragile X syndrome is recommended for early diagnosis and intervention of potential genetic diseases, especially for those high-risk families.

The genetic background of one disease varies much across China with a large population and 56 ethnicities. Rich dada about various genetic diseases have accumulated in medical institutions around China. However, a large-scale or state-level biological sample database has not been built up. Current research on genetic diseases is usually limited in Jiangsu and Anhui Provinces, China. Multi-center cohort studies of genetic diseases should be conducted in the future to further improve the diagnosis and treatment of neurogenetic diseases.

## Limitation

Some limitations in this study should be noted. First, all GDD/ID children were from Nanjing and surrounding regions. Second, they were all Han Chinese and we did not recruited subjects an ethnic minority. Our findings should be further validated in the large-scale study involving multiple ethnic minorities.

## Data availability statement

The raw sequence data reported in this paper have been deposited in the Genome Sequence Archive (Genomics, Proteomics & Bioinformatics 2021) in National Genomics Data Center (Nucleic Acids Res 2022), China National Center for Bioinformation/Beijing Institute of Genomics, Chinese Academy of Sciences (GSA-Human: HRA003395) that are publicly accessible at https://ngdc.cncb.ac.cn/gsa-human.

## Ethics statement

The studies involving human participants were reviewed and approved by the Ethics Committee of Children's Hospital of Nanjing Medical University (202110083-1). Written informed consent to participate in this study was provided by the participants' legal guardian/next of kin.

## Author contributions

RL designed the study and helped write the manuscript. DW performed the interpreted data and wrote the manuscript. All authors contributed to the article and approved the submitted version.
